# The Association between Systemic Immune-Inflammation Index and All-Cause Mortality in Acute Ischemic Stroke Patients: Analysis from the MIMIC-IV Database

**DOI:** 10.1155/2022/4156489

**Published:** 2022-08-02

**Authors:** Shaosheng Wu, Xiaoting Shi, Quan Zhou, Xiangjie Duan, Xiongfei Zhang, Huajing Guo

**Affiliations:** ^1^Department of Anesthesiology, First People's Hospital of Changde City, Changde, Hunan, China; ^2^Department of Science and Education Section, First People's Hospital of Changde City, Changde, Hunan, China; ^3^Department of Infectious Diseases, First People's Hospital of Changde City, Changde, Hunan, China

## Abstract

**Purpose:**

Acute ischemic stroke (AIS) is a devastating disease and remains the leading cause of death and disability. This retrospective study aims to investigate associations between systemic immune-inflammation index (SII) and all-cause mortality in patients with AIS. *Patients and Methods*. We used the data from Medical Information Mart for Intensive Care IV. A total of 1,181 patients with acute ischemic stroke (AIS) were included. Systemic immune-inflammation index (SII) was calculated as platelet count (/L) × neutrophil count (/L)/lymphocyte count (/L). The main outcomes were 30-day all-cause mortality. The association between SII with mortality was evaluated using the Cox proportional hazards regression model.

**Results:**

After adjusting for potential covariates, the highest quartiles of SII versus the lowest quartiles of SII, the HR was 2.74 (CI 1.79–4.19, *P* < 0.001). Log-transformed SII was significantly associated with 30-day all-cause mortality (HR 2.44; CI 1.72–3.46, *P* < 0.001). Furthermore, we found that there is a nearly linear relationship (*P*=0.265) between logarithmic transformed SII with all-cause mortality.

**Conclusion:**

Elevated SII of patients with acute ischemic stroke increased the risk of 30-day all-cause mortality. SII may serve as a useful marker to elucidate the role of thrombocytosis, inflammation, and immunity interaction in the development of AIS.

## 1. Introduction

Acute ischemic stroke (AIS) is a devastating disease; it leads to high morbidity and mortality. In mainland China, the in-hospital death rate was 1.9% for stroke patients [1]. The Ministry of Health China Stroke Prevention Project Committee (CSPPC) has established a stroke center network, stroke map, and stroke “Green Channel” to create three 1 h gold rescue circles, which led to a significant improvement in stroke [1, 2]. But this disease still causes us to suffer huge pain and losses.

The mechanism of AIS is complex and needs more investigation. Scientific evidence has revealed that immunity and inflammation play crucial roles in AIS, which are the main causes of the most cardiovascular event [3, 4]. Growing evidence from prospective cohort studies has shown that differential leukocyte counts, including counts of lymphocytes [5], neutrophils [6, 7], and other granulocyte [5], are associated with the risk of cardiovascular disease. A study has previously found that platelet counts were significantly associated with stroke events [8]. Emerging data have also shown that some inflammation and immune indices based on the complete blood count, such as platelet-lymphocyte ratio (PLR) and neutrophil-lymphocyte ratio (NLR), can serve as predictors of cardiovascular events in patients at risk of primary cardiovascular disease [9, 10]. However, there is a novel inflammation and immune marker, defined as a systemic immune-inflammation index (SII), which is calculated using platelet, neutrophil, and lymphocyte counts (*P*^*∗*^N/L). This marker was shown to reflect the severity of systemic inflammation in patients with carcinoma [11] and has high prognostic values in different types of cancer [12, 13]. SII was recently reported to be positively associated with mortality in patients with heart failure [14]. Some studies have shown that it is related to the incidence, severity, and prognosis of AIS [15, 16], but it is unknown whether it is related to the mortality of patients who stay in the intensive care unit. To explore the correlation between SII and AIS all-cause mortality, our study analyzed the data from Medical Information Mart for Intensive Care IV database.

## 2. Materials and Methods

### 2.1. Database

This study was a restrictive observation study from the Medical Information Mart for Intensive Care IV database (MIMIC-IV version 1.0) from 2008 to 2019. This database, an update to MIMIC-IV, is deidentified according to the Health Insurance Portability and Accountability Act Safe Harbor provision and has approval from the Massachusetts Institute of Technology and Institutional Review Board of Beth Israel Deaconess Medical Center (BIDMC). MIMIC-IV contains clinical information from patients in the Intensive Care Unit (ICU) at BIDMC. The author finished the Collaborative Institutional Training Initiative (CITI) program course named “Data or Specimens Only Research” and achieved access to the database.

### 2.2. Data Extraction

The Structured Query Language (SQL) with PostgreSQL (version 9.6) was introduced to extract the data from the MIMIC-IV database. Adult patients who were diagnosed with AIS based on the ninth and tenth revision of the International Classification of Diseases (ICD-9/10) code during their admissions were included in this study (ICD-9 codes: 34660, 34661, 34662, 34663, 43301, 43311, 43321, 43331, 43381, 43391, 43401, 43411, and 43491; ICD-10 code: I63). The following information was collected: general information, vital signs, scoring systems (the sequential organ failure assessment (SOFA) score, acute physiology score III (APS III) score, and simplified acute physiology score II (SAPS II)), Glasgow coma scale (GCS), comorbidities, laboratory data, and treatments. All of the laboratory results extracted were from the first test results after the patient entered the ICU. The primary outcome was 30-day all-cause mortality, and the secondary outcome was 90-day all-cause mortality.

### 2.3. Evaluation of Systemic Immune-Inflammation Index

The systemic immune-inflammation index was calculated from absolute peripheral platelet counts (*P*, ^*∗*^ 10^9^/L), neutrophil count (N, ^*∗*^10^9^/L), and lymphocyte counts (L, ^*∗*^ 10^9^/L) using the following formula: SII = *P* ×N/L [16]. The SII value was also transformed to a logarithmic scale to minimize the skewness of the underlying distribution.

### 2.4. Inclusion Criteria

**Figure 1:**
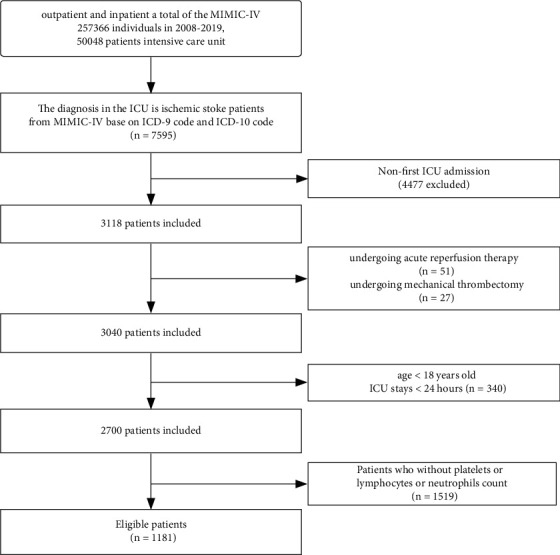
Flowchart of subject screening.

Patients from 2008 to 2019 were identified in the MIMIC-IV database. The inclusion criteria were as follows: adult patients (age, 18–89 years) with acute ischemic stroke. Exclusion criteria were as follows: nonfirst admission to ICU; age <18; a length of ICU stay less than 24 hours; missing platelet, neutrophil, or lymphocyte counts data at ICU admission; and accepted mechanical thrombectomy or intravenous thrombolysis.  Figure 1 shows the detailed flowchart.

### 2.5. Statistical Analysis

The *R* software (version 3.42) and Free Statistics software (version 1.3) were used. *P* values less than 0.05 (two-sided) were considered statistically significant. In our statistical analysis, we followed the methods of Bin Hu et al. [17]. 1,181 participants were divided into four groups according to SII quartiles calculated at baseline. Normally distributed continuous variables are presented as mean ± standard deviation, nonnormally distributed continuous variables as medians with their interquartile ranges, and categorical variables as total number and percentage. We used the chi-square test for categorical variables and the Kruskal–Wallis test for continuous variables to compare groups. To examine the link between SII and the risk of all-cause mortality in patients with AIS, we introduced three different models by the univariate and multivariate Cox proportional hazards regression model, including the unadjusted model, the minimally adjusted model, and the fully adjusted model. SII was converted into LogSII by log-transformed because of nonnormally distribution. Cumulative hazards across SII quartiles are shown using Kaplan–Meier curves. The log-rank test was used to compare the curves. Accounting for the nonlinear correlation between SII and all-cause mortality of AIS patients, we also used a generalized additive model and the smooth curve fitting (penalized spline method) to address nonlinearity. Subgroup analyzes were conducted using a stratified Cox proportional hazard regression model. To test the robustness of our results, we performed a sensitivity analysis. We divided SII into four groups as categorical variables and calculated P for the trend to verify the result of SII as the continuous variable. Missing values for all variables are less than 5%, and missing values are imputed by the median or mean. All data were analyzed using the *R* software (version 3.42) and Free Statistics software (version 1.3). *P* values less than 0.05 (two-sided) were considered statistically significant.

## 3. Results

The detailed process of patient selection is shown in Figure 1. A total of 3118 patients satisfied the diagnostic criteria of AIS, and of these, 1181 patients fulfilled the inclusion criteria for the study.  Table 1 provides the baseline characteristics of the enrolled patients. SII was divided into quartiles based on the distribution of baseline SII in patients (Q1: <667.5, Q2 : 667.6–1243.2, Q3: 1243.3–2242.0, and Q4: >2242). The baseline characteristics divided by SII are given in Table 1. According to Table 1, we found that there are significant differences in age, heart rate, respiratory rate, hyperlipidemia, COPD, paraplegia, neutrophils, lymphocytes, platelets, WBC, RBC, hemoglobin, RDW, anion gap, calcium, chloride, BUN, glucose, ALT, AST, INR, PT, PTT, NOAC, mechanical ventilation, PEGJ, APS III, SAPS II, OASIS, ICU.LODS, SOFA, GCSMIN, LOS.ICU, and LOS.hospital.

Table 2 provides the results of the univariate Cox regression analysis. Through the results, the covariates included age, heart rate, respiratory rate, Charlson comorbidity, neutrophils, WBC, anion gap, bicarbonate, warfarin, NOAC, antiplatelet agents, PEGJ, mechanical ventilation, APS III, SAPS II, OASIS, SOFA, GCSMIN, HASBLED, ICU.LODS, LOS.ICU, and LOS.hospital were associated with 30-day all-cause mortality.

Table 3 provides the results of the association analysis between SII and all-cause mortality for 30-day and 90-day. Data are expressed as the hazard ratio (HR) and 95% confidence interval (CI). Log-transformed SII was described as a risk factor for AIS patients (30-day: nonadjusted model 2.35 (1.63–3.37), minimally adjusted model 2.51 (1.76–3.59), and fully adjusted model 2.43 (1.72–3.46); 90-day: nonadjusted model 2 (1.56–3.11), minimally adjusted model 2.42 (1.72–3.41), and fully adjusted model 1.92 (1.36–2.72)). Furthermore, compared to low SII (Q1 group), higher SII (Q4 group) was associated with an increased risk of 30-day all-cause mortality (nonadjusted model: 2.28 (1.51–3.43); minimally adjusted model: 2.56 (1.69–3.86); and fully adjusted model: 2.74 (1.79–4.19)). On 90-day mortality, Q4 group expressed such results (nonadjusted model: 2.14 (1.44–3.17); minimally adjusted model: 2.45 (1.65–3.64); and fully adjusted model: 2.63 (1.74–3.96)). A similar trend was found in the Q2 group and Q3 group.

**Figure 2:**
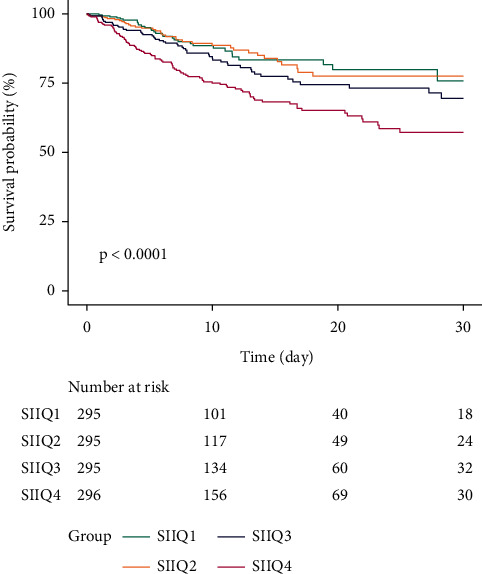
Kaplan–Meier survival curve for quartiles of SII.

The Kaplan–Meier curve for the SII quartile is shown in Figure 2. The figure indicates that survival rates of groups Q1 and Q2 were higher than groups Q3 and Q4, even though group Q4 described lowest survival probability at the time point of 30-day. Additionally, we plotted the survival rates at the time point of 30-day and 90-day (in Supplementary material 1).

**Figure 3:**
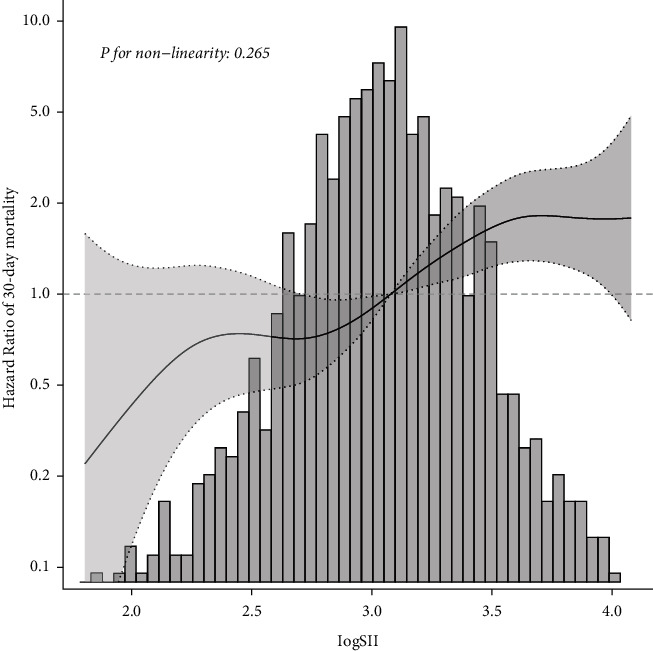
Smooth curve fitting for LogSII to HR of 30-day mortality.

We did not find an obvious nonlinear relationship between LogSII and 30-day mortality after adjusting for age, sex, heart rate, respiratory rate, Charlson comorbidity, anion gap, bicarbonate, glucose, warfarin, NOAC, antiplatelet agents, PEGJ, mechanical ventilation, SOFA, and LOS.ICU.  Figure 3 shows that the association between LogSII and 30-day mortality of AIS patients was nearly linear (*P*=0.265).

**Figure 4:**
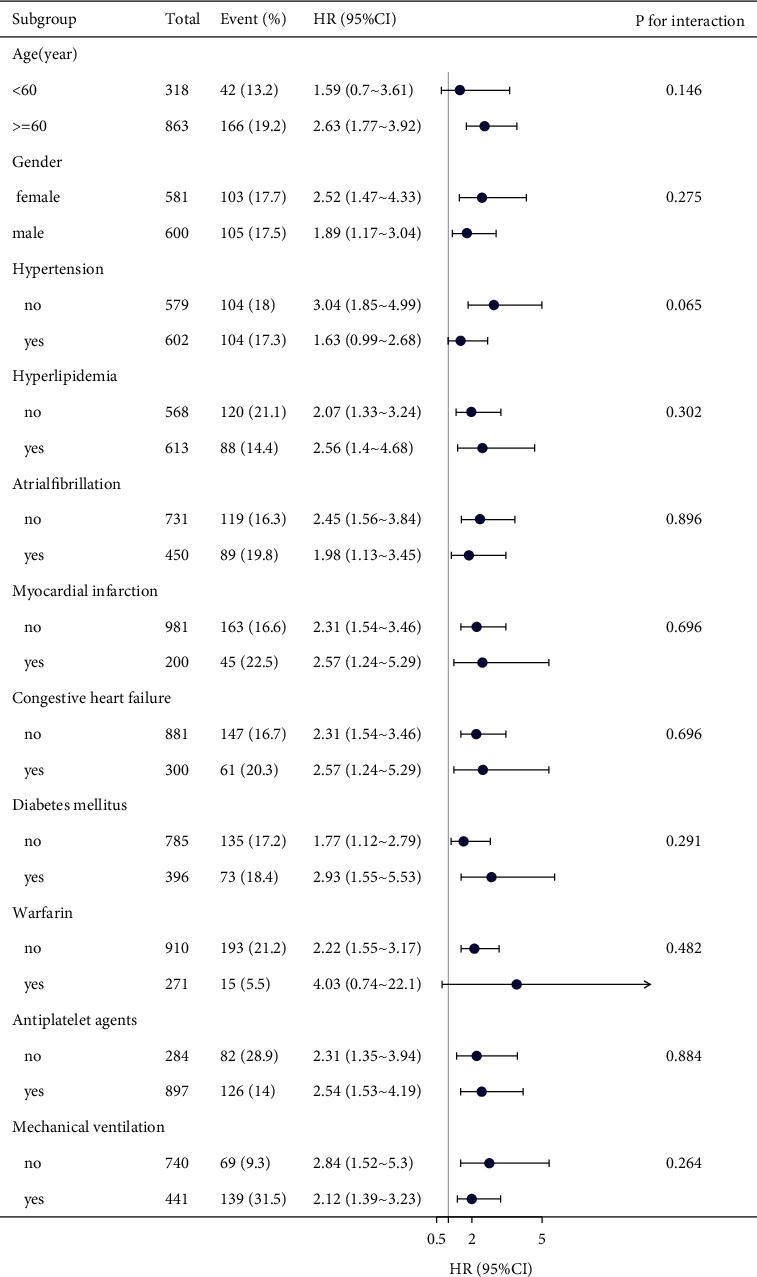
Subgroup analysis of the associations between SII and 30-day mortality.

Figure 4 shows the results of subgroup analyzes regarding the outcome of 30-day mortality. The interactions tests were not statistically significant for age, sex, hypertension, hyperlipidemia, atrial fibrillation, myocardial infarction, congestive heart failure, diabetes mellitus, warfarin, antiplatelet agents, or mechanical ventilation use (P for interaction = 0.146, 0.275, 0.065, 0.302, 0.896, 0.696, 0.696, 0.291, 0.482, 0.884, and 0.264).

## 4. Discussion

In this retrospective study, we found that SII was positively correlated with all-cause mortality in AIS patients using the unadjusted and adjusted Cox regression models. In particular, groups with a higher SII expressed higher mortality. We also found that the relationship between LogSII and 30-day mortality of patients with AIS was nearly linear. These results indicate that SII may be a new predictor of mortality of AIS.

As a new marker of inflammation, SII was proved to be used for the prediction of tumor prognosis prediction [12]. In recent years, some correlation with AIS is due to the mechanisms of immunity, inflammation, and atherosclerosis [18, 19]. SII integrates neutrophils, platelets, and lymphocytes, which cover the three aspects of inflammation, immunity, and thrombosis. Neutrophils, which are systemic inflammation markers, infiltrate the ischemic brain within 30 minutes to a few hours [20]; it releases several cytokines and proinflammatory mediators such as inducible nitric oxide synthase and matrix metalloproteinases to contribute to inflammation in the lesion [21]. Neutrophils also participate in the early destruction of the blood-brain barrier [22]. As one hallmark of brain ischemic injury, clinical studies have shown that the infiltration of neutrophils after ischemic stroke was correlated with the severity of the injury [23, 24]. At the time of ischemic stroke, platelets arrive at the injured vasculature in the brain first. Except for thrombosis, platelets can directly interact with circulatory leukocytes by changing the surface expression of P-selection or CD40 to form platelet-leukocyte aggregates activating the innate immune response to ischemia. Platelets also mediate dendritic cells antigen presentation to T cells. Platelets form heterotypic aggregates with neutrophils as a function of TLR7, TLR2, and TLR4 activation in the circulation [25]. The role of lymphocytes in the pathogenesis of AIS remains controversial. Lymphocytes are elevated in the ischemic brain later than neutrophils (3–6 days after AIS) [26]. Lymphocytes are a prognostic marker for cardiovascular disease. Several studies reported that lymphocytes play an important role in the healing and repair effects of inflammation [27]. Lymphocytes including B and T cells, especially CD4+, CD8+ T cells, and *γδ*T cells, can produce proinflammatory cytokines such as interferon-*γ* and IL-17; however, Treg cell (CD4+CD25+Foxp3+Treg cell) is beneficial for inflammation by releasing anti-inflammatory cytokines such as IL-10 that are neuroprotective through the IL-10/JAK/STAT, PI3K and MAPK pathway [24, 28].

In our study, the high-level SII group was found to have higher neutrophils, platelets, and lower lymphocytes, which is consistent with our previous hypothesis. In addition, we performed a sensitivity analysis to determine the stability of our results and found no interaction among covariate subgroups, which may require larger sample sizes to verify.

Our study has some strengths. A key finding was that our study was the first study to reveal the association of SII with 30-day mortality in patients with AIS. Second, this study enrolled 1,181 patients, which is a large sample size for the clinical study of AIS. Third, we explored both the linear and nonlinear relationships. Fourth, we analyzed the exposure variable (SII) as not only a continuous variable but also a categorical variable and calculated the hazard ratio using Cox regression models. Such a method can minimize the incidence of contingency in statistical analysis and enhance the reliability of the final results.

There are some limitations to our study. First, SII was calculated using only the first test results after the patient entered the ICU. The optimal time point needs to be explored. Second, our study lacked some information at the time of participants' blood sample collection, including indicators of the acute inflammatory state (i.e., CRP, IL-1, IL-6, and TNF-*α*) [29–31], some other drugs use such as the use of steroids and nonsteroidal anti-inflammatory drugs, previous use of antibiotics and therapy durations, and information about other diseases that could cause inflammation, such as pneumonia, all of which may influence baseline leukocyte counts. Finally, as a single-center retrospective study, selection bias was inevitable; therefore, prospective studies are needed to confirm this finding.

## 5. Conclusions

SII is positively correlated with 30-day mortality in patients with AIS.

## Figures and Tables

**Table 1:** Baseline characteristics of participants by quartiles of the systemic immune-inflammation index (*N*  = 1181).

Variables	Total	Quartiles of the systemic immune-inflammation index (10^9^/L)				*P* value
		Q1	Q2	Q3	Q4	
		<667.5	667.5–1243.2	1243.2–2242	>2242	
Participants (*N*)	1181	295	295	295	296	

Characteristics						
Age, years	69.1 ± 15.6	71.2 ± 15.1	69.3 ± 16.5	67.7 ± 15.0	68.2 ± 15.8	0.035
Female, *n* (%)	581 (49.2)	144 (48.8)	146 (49.5)	151 (51.2)	140 (47.3)	0.82
Heart rate	83.7 ± 18.7	78.8 ± 16.5	81.7 ± 17.7	84.4 ± 19.3	89.7 ± 19.2	<0.001
MAP, mmHg	76.3 ± 19.3	75.8 ± 18.6	75.2 ± 18.8	78.2 ± 21.0	76.2 ± 18.6	0.282
Respiratory rate	19.2 ± 5.6	18.3 ± 5.0	18.5 ± 4.8	19.7 ± 6.1	20.4 ± 6.0	<0.001
Temperature (°C)	36.8 ± 0.8	36.7 ± 0.7	36.8 ± 0.7	36.8 ± 0.7	36.8 ± 0.9	0.117
SPO_2_%	97.2 ± 3.7	97.8 ± 2.7	97.3 ± 3.8	96.9 ± 3.8	96.8 ± 4.2	0.003

Comorbidities, *n* (%)						
Hypertension, *n* (%)	602 (51.0)	152 (51.5)	158 (53.6)	145 (49.2)	147 (49.7)	0.701
Hyperlipidemia, *n* (%)	613 (51.9)	170 (57.6)	165 (55.9)	160 (54.2)	118 (39.9)	<0.001
Atrial fibrillation, *n* (%)	450 (38.1)	98 (33.2)	115 (39)	119 (40.3)	118 (39.9)	0.252
Myocardial infarct, *n* (%)	200 (16.9)	43 (14.6)	50 (16.9)	52 (17.6)	55 (18.6)	0.607
CHF, *n* (%)	300 (25.4)	68 (23.1)	69 (23.4)	86 (29.2)	77 (26)	0.291
PVD, *n* (%)	134 (11.3)	36 (12.2)	33 (11.2)	31 (10.5)	34 (11.5)	0.933
Dementia, *n* (%)	59 (5.0)	18 (6.1)	20 (6.8)	8 (2.7)	13 (4.4)	0.102
CPD, *n* (%)	233 (19.7)	48 (16.3)	45 (15.3)	72 (24.4)	68 (23)	0.007
Rheumatoid disease, *n* (%)	35 (3.0)	8 (2.7)	10 (3.4)	5 (1.7)	12 (4.1)	0.373
Peptic ulcer disease, *n* (%)	18 (1.5)	6 (2)	2 (0.7)	4 (1.4)	6 (2)	0.475
Diabetes mellitus, *n* (%)	396 (33.5)	109 (36.9)	100 (33.9)	94 (31.9)	93 (31.4)	0.751
Paraplegia, *n* (%)	592 (50.1)	141 (47.8)	163 (55.3)	159 (53.9)	129 (43.6)	0.015
Malignancy, *n* (%)	85 (7.2)	19 (6.4)	20 (6.8)	21 (7.1)	25 (8.4)	0.796
Severe liver disease, *n* (%)	17 (1.4)	7 (2.4)	3 (1)	4 (1.4)	3 (1)	0.437
Renal disease, *n* (%)	228 (19.3)	61 (20.7)	56 (19)	55 (18.6)	56 (18.9)	0.922
Metastatic solid tumor, *n* (%)	39 (3.3)	4 (1.4)	9 (3.1)	15 (5.1)	11 (3.7)	0.084
Charlson comorbidity	7.1 ± 2.8	7.2 ± 2.7	7.1 ± 2.7	7.2 ± 3.0	7.0 ± 2.8	0.787

Laboratory						
Neutrophils, 10^9^/L	8.7 ± 4.4	5.4 ± 2.8	7.2 ± 2.9	9.7 ± 3.6	12.7 ± 4.3	<0.001
Lymphocytes, 10^9^/L	1.6 ± 0.9	2.3 ± 1.1	1.7 ± 0.7	1.3 ± 0.7	1.0 ± 0.5	<0.001
Platelets, 10^9^/L	226.0 ± 89.2	180.9 ± 77.7	215.4 ± 71.4	223.0 ± 83.2	284.5 ± 90.7	<0.001
WBC, 10^9^/L	10.0 (7.7, 13.6)	7.7 (6.2, 10.1)	8.9 (7.5, 11.1)	11.1 (8.8, 13.8)	13.8 (10.5, 17.6)	<0.001
RBC, mean ± SD	4.1 ± 0.8	4.0 ± 0.8	4.1 ± 0.8	4.2 ± 0.8	4.2 ± 0.8	0.008
Hemoglobin, g/L	12.3 ± 2.4	11.9 ± 2.4	12.3 ± 2.4	12.5 ± 2.3	12.3 ± 2.4	0.032
RDW (%)	14.3 ± 1.9	14.1 ± 1.9	14.3 ± 1.9	14.3 ± 1.8	14.6 ± 2.1	0.007
Anion gap, mmol/L	15.6 ± 4.3	14.6 ± 4.4	15.1 ± 3.7	16.1 ± 4.1	16.6 ± 4.7	<0.001
Bicarbonate, mmol/L	22.9 ± 3.9	23.1 ± 3.7	23.3 ± 3.5	22.5 ± 3.9	22.6 ± 4.5	0.05
Calcium, mg/dL	8.7 ± 0.7	8.8 ± 0.8	8.8 ± 0.6	8.6 ± 0.8	8.6 ± 0.8	<0.001
Chloride, mmol/L	103.1 ± 5.5	104.3 ± 5.2	102.9 ± 5.4	102.8 ± 5.2	102.5 ± 6.0	<0.001
Sodium, mmol/L	138.9 ± 4.5	139.5 ± 4.1	139.0 ± 4.5	138.7 ± 4.1	138.6 ± 5.4	0.098
Potassium, mmol/L	4.3 ± 0.8	4.3 ± 0.7	4.3 ± 0.8	4.3 ± 1.0	4.3 ± 0.8	0.933
Creatinine, mEq/L	1.0 (0.8, 1.3)	1.0 (0.8, 1.3)	0.9 (0.8, 1.2)	1.0 (0.8, 1.3)	1.0 (0.8, 1.4)	0.487
BUN, mg/dL	18.0 (14.0, 26.0)	18.0 (14.0, 26.0)	18.0 (12.0, 25.0)	18.0 (14.0, 26.0)	20.0 (14.5, 30.0)	0.043
Glucose, mg/dL	126.0 (104.0, 166.0)	114.0 (96.5, 143.0)	117.0 (101.0, 154.0)	127.0 (106.0, 165.5)	148.0 (119.0, 193.2)	<0.001
ALT, U/L	20.0 (14.0, 31.0)	18.0 (13.0, 26.8)	19.0 (13.0, 28.0)	21.0 (14.0, 33.0)	22.0 (16.0, 39.0)	0.001
AST, U/L	27.0 (19.0, 43.0)	25.0 (19.0, 37.0)	25.0 (19.0, 39.0)	29.0 (19.0, 45.0)	29.0 (20.0, 53.0)	0.002
INR	1.1 (1.0, 1.3)	1.1 (1.0, 1.3)	1.1 (1.0, 1.3)	1.2 (1.1, 1.3)	1.1 (1.1, 1.3)	0.02
PT seconds	12.5 (11.4, 14.4)	12.4 (11.3, 14.5)	12.2 (11.3, 13.9)	12.8 (11.6, 14.6)	12.7 (11.7, 14.4)	0.002
PTT seconds	28.8 (25.7, 32.1)	29.8 (26.2, 33.0)	28.4 (25.7, 32.0)	28.7 (25.3, 33.0)	28.3 (25.7, 31.8)	0.031

Drugs use and treatment, *n* (%)						
Warfarin, *n* (%)	271 (22.9)	60 (20.3)	67 (22.7)	75 (25.4)	69 (23.3)	0.534
NOAC, *n* (%)	152 (12.9)	44 (14.9)	45 (15.3)	39 (13.2)	24 (8.1)	0.035
Antiplatelet agents, *n* (%)	897 (76.0)	221 (74.9)	224 (75.9)	236 (80)	216 (73)	0.235
MV, *n* (%)	441 (37.3)	95 (32.2)	87 (29.5)	111 (37.6)	148 (50)	<0.001
PEGJ, *n* (%)	88 (7.5)	7 (2.4)	18 (6.1)	22 (7.5)	41 (13.9)	<0.001

Scoring systems						
APS III	48.4 ± 24.4	43.1 ± 22.4	44.3 ± 22.0	47.3 ± 22.8	58.9 ± 27.0	<0.001
SAPS II	34.8 ± 13.4	33.4 ± 13.0	33.3 ± 13.7	34.4 ± 12.7	38.0 ± 13.7	<0.001
OASIS	33.8 ± 9.3	31.8 ± 8.9	32.7 ± 9.1	33.6 ± 8.8	37.2 ± 9.6	<0.001
LODS days	4.0 (2.0, 7.0)	4.0 (1.5, 6.0)	4.0 (2.0, 6.0)	4.0 (2.0, 7.0)	5.0 (3.0, 8.0)	<0.001
SOFA	4.0 (2.0, 7.0)	4.0 (2.0, 6.0)	4.0 (2.0, 6.0)	4.0 (2.0, 6.5)	5.0 (3.0, 7.0)	<0.001
GCS	10.8 ± 3.8	11.5 ± 3.6	11.2 ± 3.6	10.8 ± 3.8	9.5 ± 4.0	<0.001
HASBLED	1.4 (1.0, 1.8)	1.0 (1.0, 2.0)	1.0 (1.0, 2.0)	1.0 (1.0, 2.0)	1.0 (1.0, 2.0)	0.332
LOS.ICU	3.7 (1.9, 7.4)	3.1 (1.8, 6.1)	3.2 (1.9, 6.8)	3.8 (2.0, 8.3)	4.4 (2.3, 9.1)	<0.001
LOS.hospital	8.7 (4.8, 15.8)	7.3 (4.0, 13.2)	8.3 (4.6, 14.8)	9.0 (5.2, 17.4)	11.2 (5.5, 18.8)	<0.001

Death, *n* (%)						
ICU mortality, *n* (%)	134 (11.3)	21 (7.1)	20 (6.8)	40 (13.6)	53 (17.9)	<0.001
30-day mortality, *n* (%)	208 (17.6)	31 (10.5)	35 (11.9)	53 (18)	89 (30.1)	<0.001
90-day mortality, *n* (%)	226 (19.1)	34 (11.5)	38 (12.9)	61 (20.7)	93 (31.4)	<0.001

Data were expressed as mean ± SD/median (interquartile ranges (IQR)) for continuous variables and percentage for categorical variables. SD, standard deviation; IQR, interquartile range; MAP, mean arterial pressure; SPO_2_, saturation of percutaneous oxygen; CHF, congestive heart failure; PVD, peripheral vascular disease; CPD, chronic pulmonary disease; WBC, white blood cell; RBC, red blood cell; RDW, red blood cell distribution width; BUN, blood urea nitrogen; ALT, alanine aminotransferase; AST, aspartate aminotransferase; INR, international normalized ratio; PT, prothrombin time; PTT, partial thromboplastin time; MV, mechanical ventilation; PEGJ, percutaneous gastrojejunostomy; NOAC, new oral anticoagulants; APS III, acute physiology score III; SAPS II, simplified acute physiology score; OASIS, Oxford acute severity of illness score; LODS, logistic organ dysfunction score; SOFA, sequential organ failure assessment; GCS, Glasgow coma scale; LOS.ICU, length of stay in the ICU; LOS.hospital, length of stay in the hospital.

**Table 2:** Univariate Cox regression analysis.

Item	HR (95% CI)	*P*
Gender: male vs. female	0.92 (0.7, 1.21)	0.567
Age, years	1.02 (1.01, 1.03)	<0.001
Heart rate	1.01 (1.01, 1.02)	<0.001
MAP, mmHg	0.9968 (0.9897, 1.004)	0.383
Respiratory rate	1.05 (1.02, 1.07)	<0.001
Temperature (°C)	0.97 (0.82, 1.14)	0.679
SPO_2_	0.99 (0.95, 1.02)	0.357
Hypertension	1.13 (0.86, 1.49)	0.371
Hyperlipidemia	0.76 (0.58, 1)	0.051
Atrial fibrillation	1.13 (0.86, 1.49)	0.379
Myocardial infarct	1.2 (0.86, 1.67)	0.281
Congestive heart failure	0.95 (0.71, 1.29)	0.755
Charlson comorbidity	1.07 (1.02, 1.12)	0.006
Neutrophils (10^9^/L)	1.08 (1.05, 1.11)	<0.001
Lymphocytes (10^9^/L)	0.87 (0.74, 1.03)	0.1
Platelets (10^9^/L)	1.0003 (0.9989, 1.0018)	0.638
WBC (10^9^/L)	1.05 (1.03, 1.07)	<0.001
Hemoglobin (g/L)	0.9982 (0.9455, 1.0539)	0.949
RDW (10^9^/L)	1.02 (0.95, 1.08)	0.624
BUN (mg/dL)	1.0043 (0.9988, 1.0099)	0.124
Anion gap (mmol/L)	1.05 (1.03, 1.08)	<0.001
Bicarbonate (mmol/L)	0.95 (0.92, 0.98)	0.002
Chloride (mmol/L)	0.9955 (0.9733, 1.0181)	0.692
Sodium (mmol/L)	1.0051 (0.9781, 1.0327)	0.716
Potassium (mmol/L)	0.93 (0.79, 1.09)	0.346
Glucose (mg/dL)	1.0018 (1.0008, 1.0028)	<0.001
INR	0.95 (0.78, 1.17)	0.659
PT seconds	0.9963 (0.9763, 1.0167)	0.719
PTT seconds	1.0036 (0.9964, 1.0108)	0.33
Warfarin	0.2 (0.12, 0.34)	<0.001
NOAC	0.1 (0.03, 0.3)	<0.001
Antiplatelet agents	0.46 (0.35, 0.61)	<0.001
PEGJ	0.1 (0.03, 0.31)	<0.001
Mechanical ventilation	2.49 (1.86, 3.33)	<0.001
APS III	1.02 (1.02, 1.02)	<0.001
SAPS II	1.04 (1.03, 1.05)	<0.001
OASIS	1.07 (1.05, 1.08)	<0.001
LODS (days)	1.16 (1.12, 1.2)	<0.001
SOFA	1.09 (1.06, 1.13)	<0.001
GCSMIN	0.89 (0.86, 0.92)	<0.001
LOS.ICU	0.94 (0.92, 0.97)	<0.001
LOS.hospital	0.81 (0.78, 0.84)	<0.001

HR, hazard ratio; CI, confidence interval; MAP, mean arterial pressure; SPO_2_, saturation of percutaneous oxygen; WBC, white blood cell; RDW, red blood cell distribution width; BUN, blood urea nitrogen; INR, international normalized ratio; PT, prothrombin time; PTT, partial thromboplastin time; NOAC, new oral anticoagulants; PEGJ, percutaneous gastrojejunostomy; APS III, acute physiology score III; SAPS II, simplified acute physiology score; OASIS, Oxford acute severity of illness score; LODS, logistic organ dysfunction score; SOFA, sequential organ failure assessment; GCS, Glasgow coma scale; LOS.ICU, length of stay in the ICU; LOS.hospital, length of stay in the hospital.

**Table 3:** Multivariate Cox regression analysis between quartiles of the systemic immune-inflammation index and 30-day mortality.

Variable	Nonadjusted (HR (95% CI), *P*)	Adjusted I (HR (95% CI), *P*)	Adjusted II (HR (95% CI), *P*)
30-day SII (quartile)			
Q1	1 (Ref)	1 (Ref)	1 (Ref)
Q2	1.03 (0.63–1.67), 0.91	1.08 (0.66–1.75) 0.765	1.3 (0.79–2.12) 0.297
Q3	1.42 (0.91–2.21), 0.123	1.61 (1.03–2.51) 0.037	1.86 (1.18–2.93) 0.007
Q4	2.28 (1.51–3.43), <0.001	2.56 (1.69–3.86) <0.001	2.72 (1.78–4.17) <0.001
Log SII	2.35 (1.63–3.37), <0.001	2.51 (1.76–3.59) <0.001	2.43 (1.71–3.45) <0.001

90-day SII (quartile)			
Q1	1 (Ref)	1 (Ref)	1 (Ref)
Q2	1 (0.63–1.58), 0.99	1.04 (0.65–1.65), 0.884	1.25 (0.78–2), 0.355
Q3	1.45 (0.95–2.21), 0.081	1.68 (1.1–2.57), 0.016	1.87 (1.22–2.87), 0.004
Q4	2.14 (1.44–3.17), <0.001	2.45 (1.65–3.64), <0.001	2.63 (1.74–3.96), <0.001
Log SII	2 (1.56–3.11), <0.001	2.42 (1.72–3.41), <0.001	1.92 (1.36–2.72), <0.001
*P* for trend	<0.001	<0.001	<0.001

Nonadjusted: no covariates were adjusted; adjusted I: we only adjusted for age and sex; adjusted II: we adjusted for age, gender, heart rate, respiratory rate, Charlson comorbidity, anion gap, bicarbonate, glucose, warfarin, NOAC, antiplatelet agents, PEGJ, mechanical ventilation, SOFA, and LOS.ICU. HR, hazard ratio; CI, confidence interval; Ref, reference.

## Data Availability

The datasets used to support this study are publicly available at https://mimic.physionet.org/.

## Supplementary Materials

Supplementary Materials In supplementary material 1, 30-day and 90-day death rate in the SII quartile were graphed and found that death outcome was mostly concentrated at the time point of 30-day.
