# Supplementary figures and images for: The Association between Systemic Immune-Inflammation Index and All-Cause Mortality in Acute Ischemic Stroke Patients: Analysis from the MIMIC-IV Database
Source: Emerg Med Int. 2022 Aug 2;2022:4156489. doi: 10.1155/2022/4156489 (PMC9363175; doi:10.1155/2022/4156489)

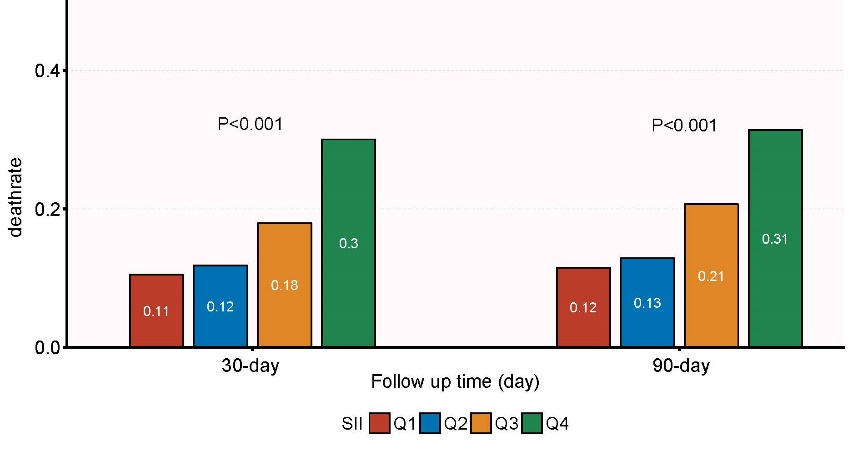

Supplement: Supplementary Materials — In supplementary material 1, 30-day and 90-day death rate in the SII quartile were graphed and found that death outcome was mostly concentrated at the time point of 30-day. [file 4156489.f1.docx]
